# A Robust Camera-Based Interface for Mobile Entertainment

**DOI:** 10.3390/s16020254

**Published:** 2016-02-19

**Authors:** Maria Francesca Roig-Maimó, Cristina Manresa-Yee, Javier Varona

**Affiliations:** Department of Mathematics and Computer Science, University of the Balearic Islands, Crta. Valldemossa km. 7.5, 07122 Palma, Spain; xisca.roig@uib.es (M.F.R.-M.); xavi.varona@uib.es (J.V.)

**Keywords:** mobile gaming, mobile entertainment, gestural interface, vision-based interface, image sensor, head-tracker

## Abstract

Camera-based interfaces in mobile devices are starting to be used in games and apps, but few works have evaluated them in terms of usability or user perception. Due to the changing nature of mobile contexts, this evaluation requires extensive studies to consider the full spectrum of potential users and contexts. However, previous works usually evaluate these interfaces in controlled environments such as laboratory conditions, therefore, the findings cannot be generalized to real users and real contexts. In this work, we present a robust camera-based interface for mobile entertainment. The interface detects and tracks the user’s head by processing the frames provided by the mobile device’s front camera, and its position is then used to interact with the mobile apps. First, we evaluate the interface as a pointing device to study its accuracy, and different factors to configure such as the gain or the device’s orientation, as well as the optimal target size for the interface. Second, we present an *in the wild* study to evaluate the usage and the user’s perception when playing a game controlled by head motion. Finally, the game is published in an application store to make it available to a large number of potential users and contexts and we register usage data. Results show the feasibility of using this robust camera-based interface for mobile entertainment in different contexts and by different people.

## 1. Introduction

Nowadays, mobile devices are equipped with different sensors such as accelerometers or gyroscopes, which allow for new sensor-based mobile interaction. Further, cameras on mobile devices can allow for vision-based interfaces, that is, interfaces which use visual information as an input to the system in a human-computer interaction (HCI) context. Mobile vision-based interfaces have been analyzed specially to detect the movement of the device [1,2], but if we take into account the front camera, the face and the head of the user can be assumed to be visible and, therefore, the user can interact with face expressions/gestures [3] or head movements [4,5,6,7].

Mobile devices are used with numerous purposes apart from communication needs. The entertainment category and the gaming subcategory are very popular in the application stores [8,9,10] and the market will accelerate in the years to come. Just as an example, the number of mobile gamers in the USA for 2014 was 147.6 million and it is expected to grow up to 209.5 million in 2019 [11]. Mobile gaming is primarily for quick entertainment, however, mobile gamers are playing more often, and for longer periods of time, a 57% more from 2012 (one hour and 20 min) to 2014 (over two hours per day) [12], so introducing new sensor-based interfaces for mobile gaming can increase the possibilities for entertainment options and make the experience more compelling.

Gesture interfaces gained in popularity for gaming purposes in video consoles thanks to systems such as the Microsoft Kinect, but in mobile devices, these interfaces have still not been really exploited. As entertainment apps using head tracking are still rare, we will focus our analysis on mobile gaming interaction based on head movements. Further, although we could find a few research works studying or comparing different aspects of head-trackers [13,14,15] and several apps which use this kind of input for accessing the mobile device [16,17] or for gaming [18,19,20,21], the system’s usability and user experience were not evaluated. Moreover, studies in HCI are often conducted in a highly controlled environment. Such studies can have a high internal validity but often lack external validity, that is, the findings can be reliable but cannot always be generalizable to other people and other situations [22]. Therefore the external validity should be pursued on two fronts: the participants involved in the study have to be representative of a larger intended population (*i.e*., study *in the large*), and the experimental environment and procedures have to be representative of real world situations where the interface will be used (*i.e*., study *in the wild*).

In this work, we describe the design and development of a camera-based interface for mobile devices and we evaluate its use, usage and the user’s perception towards it for mobile interaction. Specifically, we analyze the camera-based interface applied to an entertainment context. First, we present an evaluation in laboratory conditions to explore the effectiveness and efficiency of the system, different factors to configure the interface such as the gain or the device’s orientation, as well as the optimal target size for the interface. Then, we present a game developed for studying its usage and the user’s perception through an *in the wild* user study with 25 participants. In addition, the camera-based game is published in an application store to make it available to a large number of potential users and contexts, and we analyze the data of the users who downloaded it.

The paper is arranged as follows: Section 2 presents the related work regarding mobile head trackers and camera-based interfaces for mobile devices used in entertainment contexts. Section 3 describes the design and development of the head-tracker for mobile devices. Section 4 reports the experience in evaluating the camera-based interface when used as a pointing device. Section 5 presents the case of study of a game for users to interact with the camera-based interface and the results of the evaluation and the usage. Finally, the last section discusses the work and presents our conclusions.

## 2. Related Work

Both academia and industry are developing interfaces based on processing the images provided by the mobile device’s camera. The kind of interactions is twofold: interactions based on the mobile device’s movement and those based on detecting the movement of a user’s body part. We are interested in the latter ones focusing on head or face movement, and especially those interfaces being used for games.

### 2.1. Head Tracking in Mobile Devices

Research on head tracker interfaces based on image sensors for desktop computers is mature and has been conducted for a long time for HCI purposes [23,24,25,26]. This kind of research is now focusing on mobile devices as cameras are integrated and devices count with sufficient processing capacity to develop vision-based interfaces. In this subsection, we compile works related to face tracking in mobile devices used in human computer interfaces.

The representation of the head and the features selection for tracking (e.g., skin color or face geometry) is a key point for the tracking step. The tracking can be done detecting the face at each frame or by corresponding the face across frames, which would update the face location based on information from previous frames. Basically, the head tracking is performed by tracking facial features [4,27] or the entire face (in 2D or 3D) [7,15,28,29]. Frequently, the facial features selected to track are the eyes [27] or the nose [30] and the entire face is usually tracked based on skin color [15,30] or face detectors [7].

We also find commercial apps and projects with no related technical papers that track the head for diverse purposes such as the Smart Screen Samsung Galaxy S4 that uses the front camera to detect the position of the face and eyes to perform functions like scroll within documents, screen rotation or pause video playback. To offer accessibility to users with impairments, we find the EVA Facial Mouse app [16] or the Go Ahead project [17] that aim to use the head motion as a pointing device.

### 2.2. Head-Tracker Interfaces for Mobile Gaming

In this subsection, we compile works related to camera-based interfaces for mobile entertaining apps and works evaluating this kind of interfaces in entertainment contexts. Most games found in the application stores or used in research works, use the facial information to change the user’s viewpoint in first-person games or to steer the avatar in the game environment.

Mobile entertainment applications using camera-based interfaces are fairly new (all the ones mentioned in this work were published in the last two years), and can be downloaded from the main application stores, but no information is given about their development or usability evaluation.

Umoove’s Flying Experience for iOS was published in 2014 and uses the device’s front camera to track the user’s head to fly in a 3D environment and collect as many potion bottles as possible [18]. The Shtick Studios HeadStart game [21], which uses the Umoove face tracking technology, detects the horizontal head movements to turn a car right or left and avoid the obstacles. In both games, subtle movements are enough to control the interaction. The Head Moves Game (by Electronic Rescue Service) [20] controls the entire head/face position to move a character through a maze, so complete displacements of the whole head have to be made to move the character, and it is not sufficient to just turn slightly the head. Finally, FaceScape (by Inisle Interactive Technologies) [19] was published in 2015, and it is used in this work to explore the camera-based interface use and usage in entertaining contexts.

Research evaluating the usability of this kind of camera-based interfaces for mobile devices or head-trackers for mobile entertaining is scarce. Cuaresma and Mackenzie [13] compared accuracy and speed using a tilt-input interface and a head-tracker interface to play a game. Participants performed better with the tilt-input, but according to their comments, they enjoyed the head-tracker, although in some cases they felt head fatigue. In [14], the authors implemented a head-tracker which detected the face position and was used with different games or apps such as Pong or an image viewer. They used the histograms to identify the face, so they encountered problems with skin colored backgrounds. The work did not present quantitative data, but listed characteristics and challenges of head-trackers in mobile devices such as visualizing what the camera sees and how well it sees it, or noticing that sometimes is easier to tilt the device instead of moving it. The works evaluating this kind of interfaces are usually conducted in controlled environments such as laboratory conditions. Therefore, the findings cannot be always generalized to real users and real contexts.

## 3. Camera-Based Interface for Mobile Devices

In this section, an overview of the camera-based interface is given, and then we describe in detail each of the stages that comprise the system.

### 3.1. Interface Design and Development

To design the interface, we followed the design recommendations for camera-based head-controlled interfaces listed in [31] which summarizes design decisions to be taken into account for any interface based on a head-tracker and the different approaches researchers have used in desktop computers. Our camera-based interface is based on facial feature tracking instead of tracking the overall head or face. The selected facial feature region is the nose, because this region has specific characteristics (distinction, uniqueness, invariance and stability) to allow tracking, it is not occluded by facial hair or glasses and it is always visible while the user is interacting with the mobile device (even with the head rotated).

**Figure 1:**
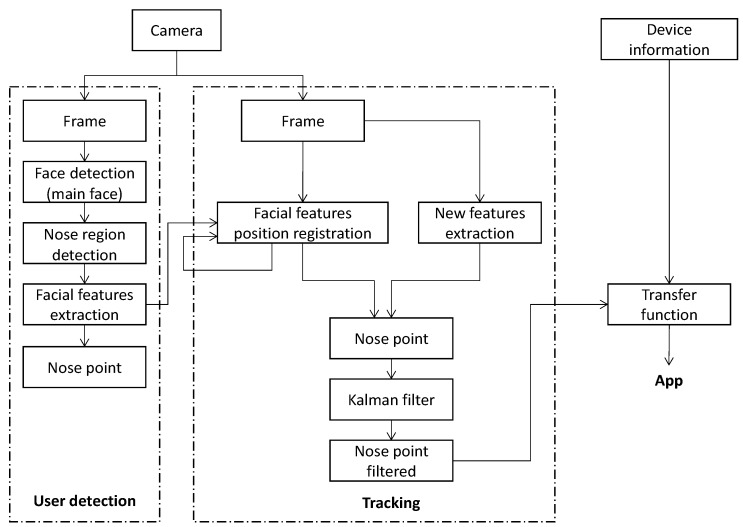
Camera-based interface design.

An overview of the interface design is depicted in Figure 1. The process is divided into two stages, the *User detection* and *Tracking*. The *User detection* stage is responsible for the processing of the initial frames coming from the camera to detect the user’s facial features to track. Once these facial features are detected, the *Tracking* stage performs their tracking and filtering. Finally, the average of all features, the nose point, is sent to a transfer function, which will use the tracking information and the device’s characteristics (e.g., screen size) to fulfill the requirements of each mobile application.

### 3.2. User Detection (and Facial Feature Selection)

**Figure 2:**
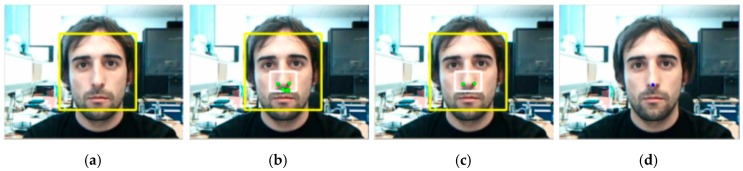
(**a**) Automatic face detection; (**b**) Initial set of features; (**c**) Feature re-selection using symmetrical constraints; (**d**) Mean of the selected features: nose point**.**

**Figure 3:**
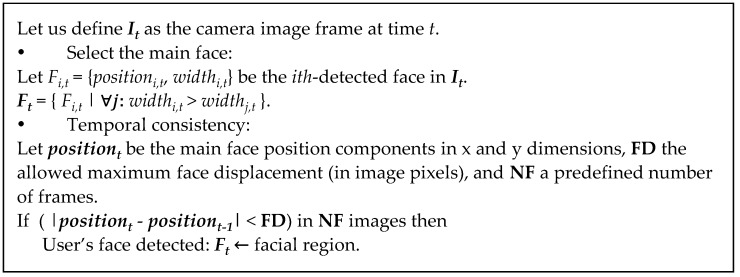
Facial region detection algorithm carried out in the *User detection* stage.

A fundamental requirement for the interface is that the user has to be automatically detected to get the control of the interaction by means of its head movements with no need of a calibration stage. Assuming that the user keeps his or her head steady for a predefined number of frames, the system automatically detects the user’s face in the image, *i.e*., the position and width of the image region corresponding to the user’s face. This facial user detection process can be based on face detection APIs integrated into the mobile platforms, which makes the capability readily available to those platforms in an optimized way. Specifically, native APIs for face detection were introduced in Android 1.5 and iOS 5. Even if different people are present in the image, the system will consider the main face (the bigger one) as the user of the system. Finally, we introduce a temporal consistency scheme in order to avoid false positives and ensure a steady user action for a proper algorithm initialization (see Figure 2a). The algorithm details to detect the user’s face region in the image are shown in Figure 3.

**Figure 4:**
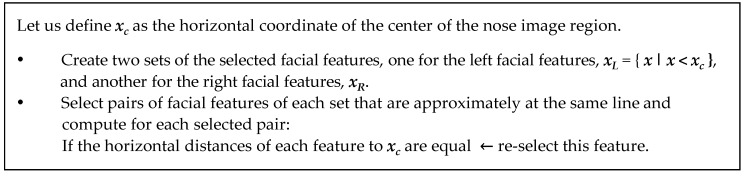
Feature re-selection algorithm for User Detection.

Observing the anthropometrical measurements of the human face, the nose region can be found in approximately the second third of the facial region. We apply this rule to select the nose image region over the facial image region detected, for searching good facial features to track. For this purpose, we use the Hessian covariance matrix over an image patch around each point of the nose image region. Generalizing, for a point u, its Hessian covariance matrix is defined such as $H=A^{T}A$. The matrix A is defined as:

$$A=\left[\begin{array}{cc} I_{x}\left(u_{1}\right) & I_{y}\left(u_{1}\right)\\ I_{x}\left(u_{2}\right) & I_{y}\left(u_{2}\right)\\ \vdots & \vdots \\ I_{x}\left(u_{N}\right) & I_{y}\left(u_{N}\right) \end{array}\right]$$

where $I_{x}$ and $I_{y}$ are the spatial partial derivatives at the image point $u_{i}$ of the neighborhood around the evaluated point u (we use a neighborhood of 10 × 10 pixels, *i.e*., N=121). In order to select an image point u as a facial feature, the eigenvalues of the Hessian matrix are computed. Let us define the facial feature quality, $\lambda_{min}$, as the minimum eigenvalue. Then, if $\lambda_{min}\;$is greater than a predefined threshold, $\lambda_{t}$, the point of the image region is selected as a good nose feature (we set $\lambda_{t}=0.01\cdot \lambda_{M}$, where $\lambda_{M}$ is the maximum value of the $\lambda_{min}$ of the entire nose region). By applying this strategy, the nostrils and the corners of the nose are found (see Figure 2b), but due to the lighting environment causing shadows, other unstable features can be detected too. The center of the nose is the ideal point to send to the transfer function. Therefore, features will be preferably placed on both sides of the nose and with certain symmetrical conditions. A re-selection of the initially found features is carried out to achieve a more robust tracking process, selecting pairs of features symmetrical respect to the vertical axis as it is described in the algorithm in Figure 4 and it is shown in Figure 2c.

Finally, the nose point is the average of all facial features being tracked, which will be centered on the nose, between the nostrils (see Figure 2d).

*The User detection* process has been evaluated using the BioID face database [32]. The BioID is a head-and-shoulder image face database, where special emphasis has been placed on real world conditions. Therefore the test set features 23 people in 1521 frontal view images considering a large variety of illumination, background, and face size. The images have been manually marked up, being the “tip of the nose” one of the feature points marked. We compared the average of the selected features for tracking, *i.e*., the nose position, given by the proposed algorithm with this annotated facial feature.

**Figure 5:**
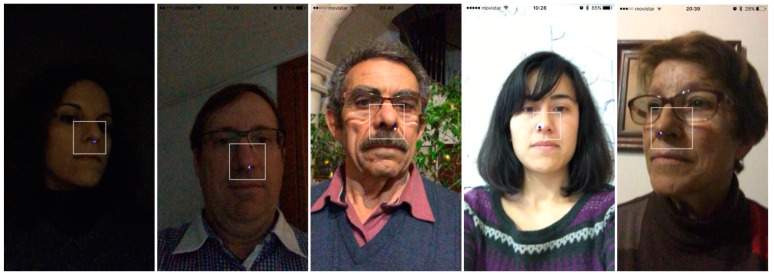
The system’s functioning with different users, lightings and backgrounds.

We used 1457 images (95.79%) as we did not consider those images where the face was not detected due to the lack of visibility of the whole face. We computed the distance between our nose position and the “tip of the nose” mark. We achieved 96.08% of nose detections with a mean of 2.34 pixels of horizontal displacement (*SD:* 2.05) and a mean of 4.98 pixels of vertical displacement (*SD:* 4.86). Errors in the nose detection are mainly due to lighting conditions that lead to different brightness on both sides of the face. The vertical displacement is bigger than the horizontal one, because nostrils are normally detected as features to track in the proposed algorithm due to the vertical symmetrical constraint. Results prove that the accuracy of the *User detection* stage is robust in real conditions. In addition, as depicted in Figure 5 in images from the front camera of the mobile device, the face and facial features detection is stable and robust for different users, light conditions and backgrounds.

### 3.3. Tracking

It is important to highlight that in the *Tracking* stage, the face does not need to be fully visible, unlike the *User detection* stage.

To track the facial features, the spatial intensity gradient information of the images is used to find the best image registration [33]. Let $u=\left(u_{x},u_{y}\right)$ be the position of a feature in a frame, the goal of feature tracking is to find the location $v=\left(u_{x}+d_{x},u_{y}+d_{y}\right)$ on the next frame assuming that the brightness of the feature does not change. In addition, to handle large motions, we use a pyramidal implementation of the classical Lucas-Kanade algorithm [34]. The tracking algorithm is robust to handle head rotation, scaling or shearing, so the user can move in a flexible way. Basically, this algorithm solves$\;\left(A^{T}A\right)\cdot d=A^{T}B$, where B is:

$$B=\left[\begin{array}{c} I_{t}\left(u_{1}\right)\\ I_{t}\left(u_{2}\right)\\ \vdots \\ I_{t}\left(u_{N}\right) \end{array}\right]$$

and $I_{t}$ is the temporal derivative at the image point $u_{i}$.

However, fast head movements can cause the lost or displacement of features to track. As we are only focusing on the nose region, when a feature is separated from the average point more than a predefined value, the feature will be discarded. When there are not enough features to track, then the *User detection* stage restarts.

To provide robustness to the *Tracking*, we recover and update the nose features used to track. We follow a typical Bayesian approach to sensor fusion, combining measurements in the representation of a posterior probability. In this case, we combine for each new frame, the tracked nose features with new detected features. For this goal, when the user's face is looking up towards the camera, we search for new features to track on the nose region. To update the set of tracked features, a probability of re-initialization is used to include the new detected features in the current nose feature set. In our case, this probability value has been set to 0.05. The user does not participate actively in this phase; he or she will just feel a subtle readjustment of the point on to the nose. Then, we apply a velocity constant Kalman filter to smooth the positions [35] (see blue point in Figure 5).

The proposed *Tracking* stage is able to run in real-time on current mobile devices with different CPU platforms. Table 1 shows the processing times in milliseconds (ms) of the head-tracker for different mobile devices when operating with the current image resolution (144 × 192). A performance test with different image resolutions in an iPhone 6 Plus platform (A8: 64 bits, 1.4 GHz ARMv8-A dual core) is also included as a comparison in Table 2.

Finally, the app using the head-tracker will decide how to proceed with the nose point given. There will be a transfer function responsible on translating the head-tracker information to the app. As an example, we will describe the transfer functions for two different apps in Section 4 and Section 5.

## 4. Evaluating the Interface as a Pointing Device

**Figure 6:**
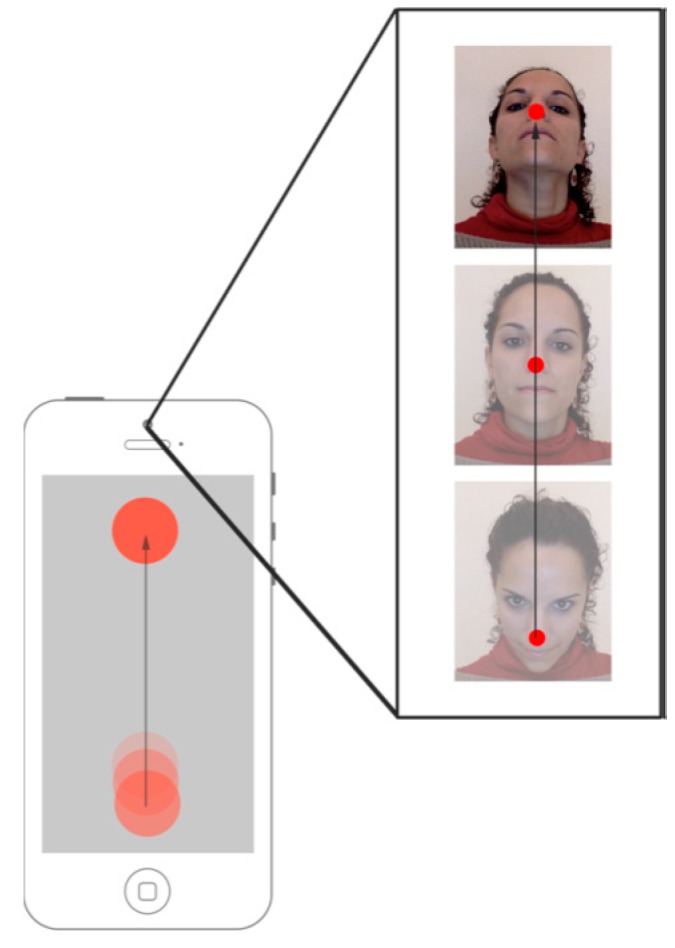
The camera-based interface processes the user’s head motion to translate it to a position in the mobile’s screen.

In this first evaluation, the nose point is mapped to the screen to provide the user with a pointing device (see Figure 6). In the next subsection, the transfer function for this app is described and then, the evaluation test is explained together with the achieved results.

### 4.1. Transfer Function

The average of all facial features, nose point, is translated to the mobile screen by means of a predefined transfer function based on differential positioning. In this approach, the interface reports the change in coordinates and the function’s given location is relative to the previous location rather than relative to a fixed origin.

The transfer function, Φ, maps the user’s nose position (the average of all tracked facial features), **v** = (*v_i_*, *v_j_*), to a device screen position, ***p*** = (*p_x_*, *p_y_*), at every time stamp t considering a scale factor (*s_x_*, *s_y_*) (see Equation (1)):

(1) $$\phi \left(p_{x},p_{y}\right)=\left(s_{x}\cdot v_{i},s_{y}\cdot v_{j}\right)$$

Specifically, the head-tracker was implemented and optimized for an iPhone 5S running iOS 8.0. The iPhone 5S has a 4-inch retina display with 1136-by-640-pixel resolution at 326 pixels per inch (*568 × 320 pt*). Apple’s point (pt.) is an abstract unit that covers two pixels on retina devices. On iPhone 5S, one point equals 1/163 inch.

To assign the best values for the scale factor (*s_x_*, *s_y_*) for users to be able to reach all screen positions in a comfortable way and considering the mobile screen width (*w*) and height (*h*) we applied Equation (2), where 55 is the image range of the user’s nose movement (in pixels):

(2) $$s_{x}=\frac{w}{55},\;\;s_{y}=\frac{h}{55}$$

Further, a gain factor is included, that is, the amount of movement on the device in response to a unit amount of movement of the user, where the velocity (m, in pixels/frame rate) is a function between the scale factor and the gain factor (see Equation (3)):

(3) m=p·gainFactor

### 4.2. Evaluation of the Interface’s Effectiveness and Efficiency

In order to evaluate the effectiveness and efficiency of the camera-based interface, an experiment was conducted to explore its performance with different target sizes (accuracy and velocity), target locations (test if all the screen positions were accessible) and influence of the gain or device orientation [30]. Due to the small screen size, we could not perform the ISO 9241-9 Multi-directional tapping task [36].

The interface was evaluated with 19 users (four females) whose ages ranged from 23 a 69 (*mean* = 38.21, *SD* = 14.05) and none of them had previous experience with this kind of interaction. The participants in the experiment were volunteers recruited from the local town and university campus.

**Figure 7:**
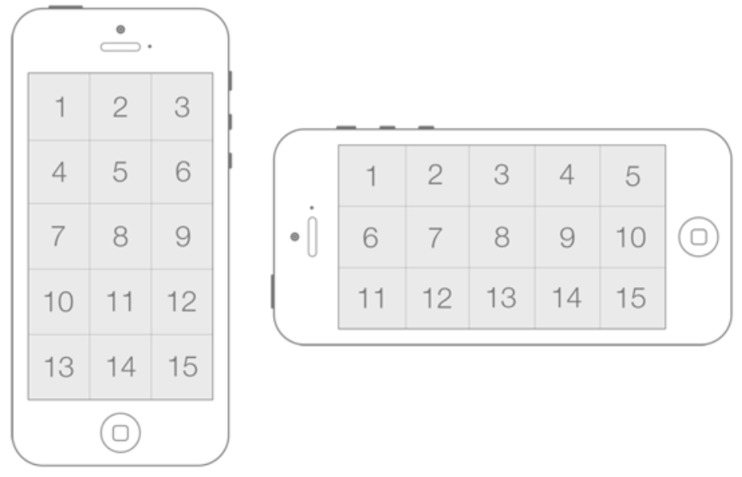
Regions of the screen in portrait and landscape right orientation.

The task to carry out was a position-select task, where the screen was divided in 15 areas (see Figure 7) whose centre was the center of the target to be selected. To perform a selection on a target, the user had to place a circular cursor inside it and tap on any part of the screen. At each moment and randomly, only one target was enabled and highlighted, and users had one chance to perform the selection. The user had a visual feedback when the cursor was inside the target, and only in this condition, the selection was considered successful; otherwise, an error was registered.

The test was conducted combining different device orientations, gains and target widths. The device orientation was an interesting factor to study, as the camera on a landscape mode is not centrally placed. The target width values were selected according to the guidelines given in the iOS Human Interface Guidelines [37,38] regarding the optimal size for tappable user interface element’s. The values were:

- Device orientation (D) = {*portrait*, *landscape right*}.
- Gain (G): {*1, 1.5*}.
- Target width (W) = {*44, 88, 106* pt}.

Each test had 12 blocks, that is, the 12 combinations of (D) × (G) × (W) with 15 trials or targets each, the first three of which were practice unbeknownst to the participant, and the order of the blocks was random. Breaks were allowed between blocks and the test duration ranged from 5.85 to 16.34 min (*mean* = 8.91 min, *SD* = 2.6 min). The total number of trials for all users was: 15 trials × 2 device orientation × 2 gain × 3 target width × 19 users = 3420.

When performing the evaluation test, users were sitting and holding the mobile phone in a comfortable position. Then, the application instructed users to proceed as accurate and fast as possible. Accuracy meant to tap as close as possible to the centre of the target.

For each target, we registered the success or error of the selection, the Euclidian distance between the effective user selection and the center of the target (accuracy) and the effective distance of the task trial divided by the movement time (velocity).

#### 4.2.1. Accuracy

**Figure 8:**
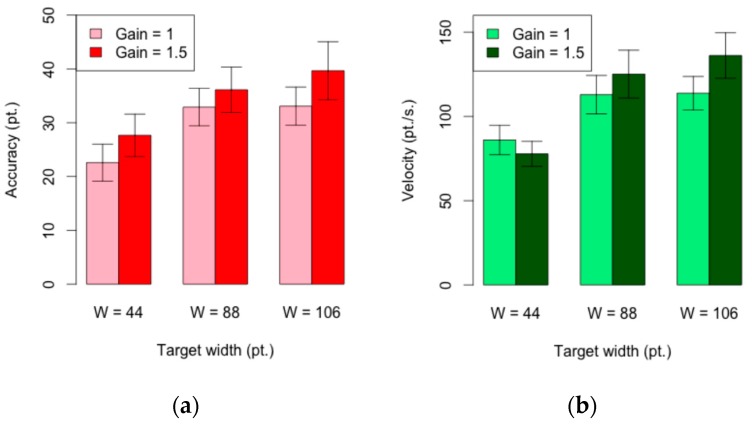
(**a**) Accuracy and (**b**) velocity by target width and gain. Error bars show 95% CI.

The mean accuracy per task was 32 points and the effect of the target width on the accuracy was statistically significant (F_2,36_ = 55.5; *p* < 0.001). The 44-target width achieved the best results in accuracy (25.12 pt) and the 106-target width was the worst (36.37 points) (see Figure 8a). Pairwise comparisons with Bonferroni correction showed that the 88 and 106-target widths were no longer significantly different *p* = 0.45 > 0.0166.

The gain had a statistically significant effect on the accuracy (F_1,18_ = 18:8; *p* < 0.001). The mean for the 1-gain was 14.38% lower than the mean of 34.48 pt. for the 1.5-gain. Finally, the effect of the device orientation on accuracy was not statistically significant (F_1,18_ = 1.32; *p* > 0.05).

#### 4.2.2. Velocity

The mean velocity per task was 108.61 pt./s. The effect of the target width on the velocity was statistically significant (F_2,36_ = 78.5; *p* < 0.001). The 44-target width achieved the lowest velocity (81.9 pt./s) and the 106-target width the highest (124.96 pt./s) (see Figure 8(b)). Pairwise comparisons with Bonferroni correction show that the 88 and 106-target widths were no longer significantly different *p* = 0.064 > 0.0166.

The effect of the gain on the velocity was statistically significant (F_1,18_ = 10.1; *p* < 0.01). The 1.5-gain = 1.5 achieved the highest velocity (113.01 pt./s.) and the 1-gain, the lowest (104.22 pt./s.). With the 44- target width the mean velocity was higher for the 1-gain that for the 1.5-gain. Finally, the effect of the device orientation on velocity was not statistically significant (F_1,18_ = 0.18, ns).

#### 4.2.3. Error Rate and Target Position 

The error rate is calculated as the erroneous selections per total number of selections. The mean per task was 0.22.

**Figure 9:**
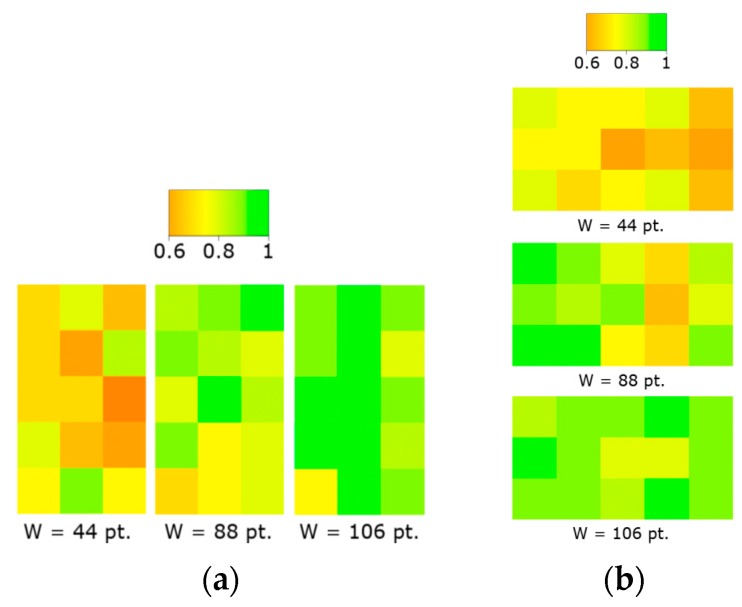
Correct selection rate by target width and with 1-gain condition for (**a**) portrait orientation and (**b**) landscape orientation.

With the 1-gain, an average of correct selection rate above 0.83 was achieved for the 88-target width (0.83) and the 106- target width (0.89). An average of correct selection rate of 0.7 was achieved for the 44-target width. There does not seem to exist a relation between the correct selection rate and the target location (see Figure 9).

## 5. Evaluation *in the wild* for Mobile Entertainment

In this section we present the FaceScape game, which uses the camera-based interface to interact with it. The game itself does not contain real innovations, but the aim was to study the usage and the user’s perception towards this kind of interaction mode in entertainment activities, where users are more willing to explore new input devices and it is a voluntary activity. Further, it was designed for short playing sessions throughout the day in a casual gaming style (each session lasted roughly one minute and a half).

**Figure 10:**
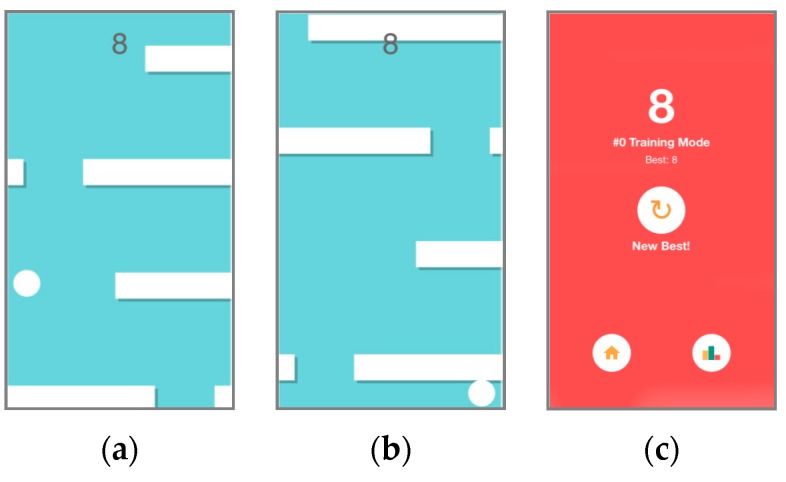
Screenshots of the game: (**a**) Ball avoiding the obstacles; (**b**) Ball being dragged down due to the collision with an obstacle; (**c**) Game lost.

We wanted to keep the game relatively simple regarding the interaction; therefore, the game just reacts to the horizontal head motion. The user has to move horizontally a ball affected by upper gravity force to avoid the obstacles that go randomly appearing (see Figure 10a). The collision with them causes the ball to be dragged down (see Figure 10b), so the game finishes when the ball crosses the bottom border as shown in Figure 10c.

The design of the game includes game mechanics to design for engagement and fun [39,40] such as points, levels or rankings. To increase the playability, the game’s difficulty is increased progressively by speeding the falling obstacles and the gravity force applied to the ball. Moreover, to motivate and engage players we show the ranking, the achieved level results and the number of played sessions.

The game has two difficulty phases: the onboarding and the challenge. The onboarding phase goes from level 0 up to 40, and is where gamers learn the rules and train with the interface to play the game. Levels are increased when an obstacle is avoided. Once level 40 is achieved for the first time, the challenge phase is unlocked and it is the default level. However, the gamer can always play the onboarding phase.

**Figure 11:**
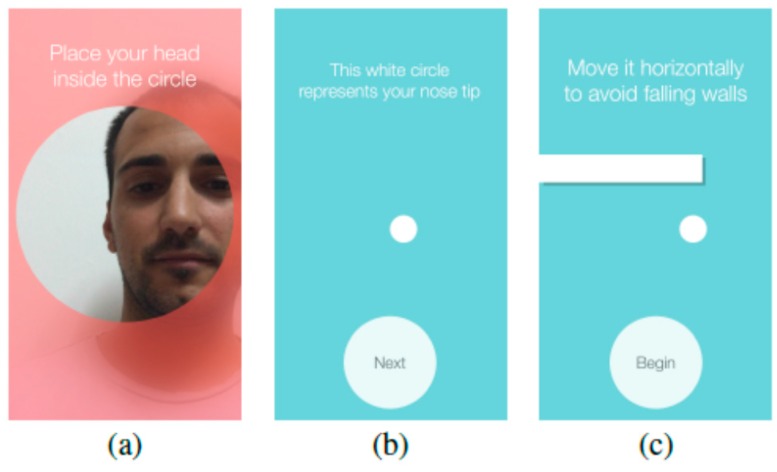
Screenshots of the instructions: (**a**) Instructions for the correct position for using the head-tracker (“Place your head inside the circle”); (**b**,**c**) Instructions explaining how to interact with the game and its objective (“The white circle represents your nose tip” “Move it horizontally to avoid falling walls”).

The game is designed to be self-explained as evaluators will not be close to the user to give explanations. While playing the onboarding phase, there are instructions at the beginning of every session, explaining the objective of the game and the correct position to use the head-tracker (see Figure 11).

In the next subsections, we describe the transfer function, and then we present the results of the evaluation of the head-tracker being used to play FaceScape. First, we report the results of a supervised evaluation done *in the wild* with 29 participants. Then, we summarize the data obtained from the users who downloaded the game from the iOS App Store.

### 5.1. Transfer Function

**Figure 12:**
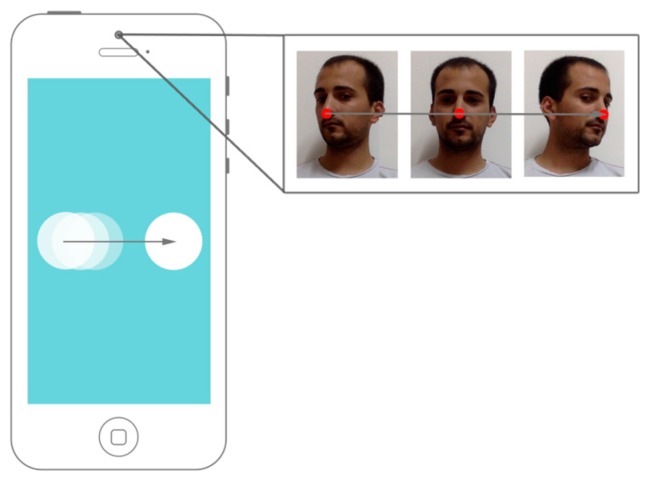
The head-tracker processes the user’s horizontal head gesture to translate it to an action on the device as a moving right gesture.

The sequence of nose points returned by the head-tracker is analyzed to detect the horizontal movement of the user’s head in both directions: left and right. The moving left head-gesture is recognized when a displacement to the right is detected on the points returned by the head-tracker (see Figure 12) and a moving left head-gesture is recognized if the displacement is in the opposite direction.

When a horizontal head-gesture is detected, the transfer function applies a horizontal impulse to a physic body to change its linear velocity without changing its angular velocity, following Newton’s second law. In our case, we just want to impart a momentum to the *x*-dimension proportional to the horizontal displacement of the head performed by the user, so a gain factor is included and our imparted momentum responds to *v_x_·gainFactor*, where the velocity (v_x_, in pixels/frame rate) corresponds to the difference in the *x*-coordinates of the nose points returned by the system in two consecutive frames.

### 5.2. Evaluation in the Wild

We conducted an *in the wild* user study of the mobile head-tracker being used with the FaceScape game before publishing it in the application store. The aim was to explore in detail how users might use and feel about this kind of input interaction method within their daily life.

The evaluation was supervised because although participants were using the game *in the wild*, we limited the length of the evaluation to a week. Further, we sent them a pre-test and post-test questionnaires, registered data (with the participant’s consent), ask them to play at least once per day and fired 6 notifications per day (scheduled at different day hours) to remind them to play and to answer several questions such as Where are you?

The study was conducted completely *in the wild* with no previous training and all the instructions were provided via email only at the beginning of the evaluation.

Twenty-five unpaid participants (eight females) with an age ranging from 21 to 69 (*mean* = 32.4, *SD* = 12.6) were recruited from Germany and Spain (52% of them from Germany) from university campuses. None of them had previous experience with head-based interaction. The only requirement to participate in the evaluation was to own an iOS device (iPhone, iPod, iPad) with iOS 8.0 installed.

#### 5.2.1. Played Sessions

During the evaluation week, a total of 2186 playing sessions were registered with an average duration of 1.46 min per session (*SD* = 0.87, *min* = 0.07, *max* = 4.97) and an average of 84.77 sessions played per participant (*SD* = 80.48, *min* = 15, *max* = 331).

By observing the number of played sessions, we get insights on the usage of the system. All the participants played more sessions than the minimum required, so the combination of the game and the camera-based interface may be engaging. Moreover, as the game was installed on their personal device, eighteen out of the twenty-five participants continued playing once the evaluation was over, registering a total of 244 sessions out of the experiment (after two weeks).

#### 5.2.2. Playing Location

**Figure 13:**
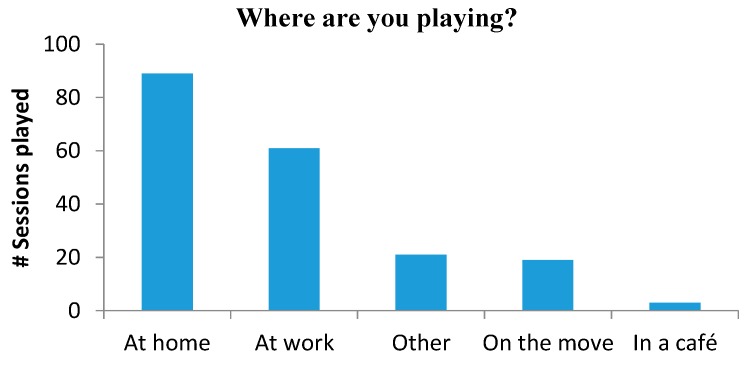
Total number of sessions played in a place.

Participants could choose to ignore or not the notification, so we registered 193 answers for the in-app questionnaires (the ones included with the daily notifications). Due to the short length of the game, participants felt comfortable playing in different contexts, even at work (see Figure 13).

A Friedman’s test revealed a significant effect in the answers for the “*Where are you?*” responses, (χ^2^(4) = 57.0, *p* < 0.001). A post-hoc test using pair-wise Wilcoxon tests with Bonferroni correction showed only significant differences for the “*At home*” answer and the rest of places (*p* < 0.001 for the three pairs).

#### 5.2.3. User’s Perception

**Figure 14:**
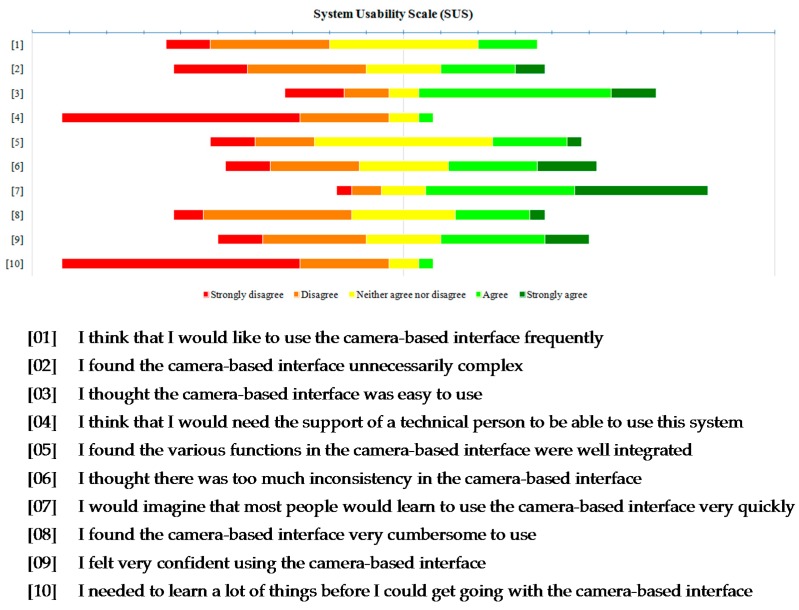
SUS questionnaire.

At the end of the evaluation, a System Usability Scale (SUS) questionnaire [41] was answered by all participants (see Figure 14). The overall average SUS score was 60.7 (*SD*: 16.4). Even though Bangor *et al*. [42] consider that systems with scores less than 70 should be considered candidates for continued improvement, this score is in the high marginal acceptability range.

Although head-trackers are a new and unknown interaction technique for mobile devices and in addition, none of the participants had previous experience with head-based interaction, the results of the SUS questionnaire showed that the head-tracker was easy to use and learn (see Figure 14). Moreover, the quantitative metrics support that evidence because all participants were able to complete the onboarding stage with an average number of attempts of 10.48 (*SD* = 9.84, *mode* = 7).

The open comments registered in the post-test questionnaire by the participants also offered information on the head-tracker and its use in this entertaining context. Not all participants found the head-tracker an interesting input device commenting “*[The interface is] not suitable for me*” or they thought “*When sitting its use is OK, but it's a little tiring when standing*”. Others did not find the system easy to use as some said that it was “*hard to control because it's slow to adapt to my movement*” or “*head-trackers are not as flexible as hand trackers*”.

However, many positive comments were given over the enjoyability of the interface “*It’s funny for games*”, “*They are spectacular, useful and funny*” or its suitability as an input device: “*I think that it could be a promising new interaction model for mobile devices*”, “*Great tool for gaming and accessibility*”, “*I think that it's a really nice system*”.

Further, people around the participant found the system intriguing, curious and surprising: “*[People] look at me interested*”, “*People were interested because they haven't seen anything alike*”, “*A friend was surprised that I could play and control a game with my head*”, “*The reaction I noticed [from the people around me] was surprise and expectation*”. Others did not understand what was happening “*People asked what was happening to me, as if my neck was aching*” or found the system funny: “*Friends think it looks funny*”, “*Many laughed, but tried to play the game too*”, “*When I played the game beside friends, they were curious and thought that it was very interesting*.”

#### 5.2.4. FaceScape in the Application Store

A version of the iOS application FaceScape which did not include the Onboarding and Challenge phases was published in the iOS App Store on 1 September 2015. With hardly any advertising, we show the usage statistics for almost three months and a half (until 14 December). It must be taken into account that these users did not know that we were evaluating the camera-based interface, so no demographic data is registered nor are questionnaires included in the app. By means of a predefined set of web services, we registered data of the app’s usage in real conditions in all the contexts, including the device, operating system, country, number of sessions played and levels achieved.

Three hundred and seven people downloaded the game (78% for iPhone, 18% for iPad and 4% for iPod). Most users were from Europe (62.9%), but downloads were also done from different regions such as Latin America and the Caribbean (12.7%), the United States and Canada (12.7%), Asia Pacific (8.8%) or Africa, Middle East, and India (2.9%). The country with most downloads was France, but users came from many different countries such as Brazil, Russia, China or Kuwait.

A total of 1191 gaming sessions were played (*mean*: 10.7, *SD*: 25.4) from 114 users. Users achieved an average level of 31, and 32.4% completed level 40. For users that played the previously so-called Challenge phase, they achieved and average level of 77 (*min*: 40, *max*: 154). These results were registered in the first three and a half months after the game’s deployment, and although more data is needed, it clearly shows how the presented camera-based interface is being used in real conditions in any context, showing its robustness as a novel interface for mobile entertainment.

### 5.3. Limitations of the Study

One of the main advantages of *in the wild* studies is their characteristic of representing the real conditions of use of the interactive systems. However, researchers lose control of the participant and some variables in the experiment. To minimize these obstacles, in our supervised study we set the evaluation period to a week, had contact by mail with the participants to send the pre and post questionnaires and sent remainders via notifications. But, it is unknown for us information on the user (e.g., how busy he or she was during the evaluation period) or a detailed description of the context where they were using the system (e.g., lighting conditions, body posture). Even more dramatic is to collect data when the game is published on an application store and people are not aware that the system is being assessed. In this last case, we just have access to the game information and data that can be automatically registered (e.g., device, country). Further, as happens with many apps [43] there is a high number of people that may download the app but will never play or will just test it and abandon it due to multiple reasons, so it is important to have a big sample of users.

## 6. Discussion and Conclusions

An extensive evaluation on how real users interact with new interfaces in mobile devices in real contexts is a requirement to ensure its robustness. This evaluation requires exhaustive studies to take into account the full spectrum of potential users and contexts, which cannot be performed only in controlled environments. Further, results in controlled environments cannot be always generalized to real users and real context.

In this work, we have described a robust camera-based interface for mobile entertainment, as these interfaces are starting to appear in games and apps. This kind of interfaces has not been really evaluated in terms of usability and user’s perception. Therefore, we gather the insights and conclusions obtained from the studies conducted.

The interface is based on detecting and tracking the nose of the user by means of processing the frames provided by the mobile device’s front camera, and its position is then used to interact with the app. We presented two evaluations: the first one, with the camera-based interface acting as a pointing device which is used to study the accuracy, different factors to configure the interface such as the gain or the device’s orientation, as well as the optimal target size for the interface. Second, we evaluated the usage and the user’s perception and impressions towards this kind of input system when playing a game controlled by head motion in a *in the wild* study to ensure real conditions.

The first evaluation demonstrated that it is feasible to interact with the interface as a pointing device to be able to reach all screen positions. Moreover, it analyzed different configuration parameters and the optimal target size to achieve the best results in accuracy. Based on this study, entertaining apps and games based on pointing could be designed to be controlled by the interface. These apps could be designed in portrait or landscape orientation, as our results demonstrated that the orientation has no effect on the accuracy. Results on the target size could be considered for the design of the playability of the game to increase the challenge of the game or to use the optimal size to design the interactive graphical elements of the app.

The second user study showed the viability of using head movements to control a game. The interface combined with the game was positively rated by users in terms of usability, especially in learnability and easiness of use. The analysis of the game’s data, reported a higher number of playing sessions than the one required in the *in the wild* user study, encouraging us to believe that the input device used with the game is engaging for users. Further, 72% of the participants continued playing after the evaluation period. Most playing sessions were carried out at home, but, users played too at work, in public places or on the move, which shows users were comfortable playing in different contexts. Moreover, the comments given by the users highlighted the amusement, the potential of these interfaces and the interest, surprise and expectation generated in their social environment.

Then, to get data from a larger group of users and maintaining the *in the wild* condition, we made the game available through the application store. Results from these users show how the presented camera-based interface is widely used by users without any instruction or training, and in very different set of contexts.

Further, a new group of users could be targeted. This kind of interface does not require contact with the device, so users with motor impairments who cannot access mobile devices though touch (e.g., due to structural deviations, mobility limitations or lack of muscle power), but count with head control, could access the mobile device. We encourage developers to consider vision based interfaces especially for implicit or short length interactions such as the ones we can find in mobile games. Further analysis in the evaluation of these interfaces has to be done regarding the fatigue and social acceptability.

## Figures and Tables

**Table 1:** Average processing time of the head-tracker for different mobile devices.

Device	CPU	Time to Process (ms)
iPad 2	A5: 32 bits, 1 GHZ, ARM Cortex-A9 dual core	251
iPhone 5s	A7: 64 bits, 1.3 GHz ARMv8-A dual core	37
iPad Air	A7: 64 bits, 1.4 GHz ARMv8-A dual core	36
iPhone 6	A8: 64 bits, 1.4 GHz ARMv8-A dual core	31

**Table 2:** Average processing time of the head-tracker with different resolutions on an iPhone 6 Plus mobile device with a CPU A8: 64 bits, 1.4 GHz ARMv8-A dual core.

Frame Height (px)	Frame Width (px)	ms
192	144	31
352	288	54
480	360	68
640	480	89

## Abbreviations

The following abbreviations are used in this manuscript:

HCI: Human Computer Interaction
